# Novel Laser-Based Obstacle Detection for Autonomous Robots on Unstructured Terrain

**DOI:** 10.3390/s20185048

**Published:** 2020-09-05

**Authors:** Wei Chen, Qianjie Liu, Huosheng Hu, Jun Liu, Shaojie Wang, Qingyuan Zhu

**Affiliations:** 1 Department of Mechanical and Electrical Engineering, Xiamen University, Xiamen 361102, China; chenwei05@stu.xmu.edu.cn (W.C.); qjliu0214@stu.xmu.edu.cn (Q.L.); jliuxmu@stu.xmu.edu.cn (J.L.); wsj@xmu.edu.cn (S.W.); 2 School of Computer Science & Electronic Engineering, University of Essex, Wivenhoe Park, Colchester CO4 3SQ, UK; hhu@essex.ac.uk

**Keywords:** autonomous robots, obstacle detection, laser point clouds, Gaussian kernel function, neural networks, 3D sensing

## Abstract

Obstacle detection is one of the essential capabilities for autonomous robots operated on unstructured terrain. In this paper, a novel laser-based approach is proposed for obstacle detection by autonomous robots, in which the Sobel operator is deployed in the edge-detection process of 3D laser point clouds. The point clouds of unstructured terrain are filtered by VoxelGrid, and then processed by the Gaussian kernel function to obtain the edge features of obstacles. The Euclidean clustering algorithm is optimized by super-voxel in order to cluster the point clouds of each obstacle. The characteristics of the obstacles are recognized by the Levenberg–Marquardt back-propagation (LM-BP) neural network. The algorithm proposed in this paper is a post-processing algorithm based on the reconstructed point cloud. Experiments are conducted by using both the existing datasets and real unstructured terrain point cloud reconstructed by an all-terrain robot to demonstrate the feasibility and performance of the proposed approach.

## 1. Introduction

With the advancement of technology, wheeled mobile robots have gradually moved towards automation and intelligence in recent years [1,2]. Mobile robots used for rescue and space exploration, etc. operate in dynamic and unstructured environments and face huge challenges due to the inherent uncertainties and the unpredictable conditions [3]. To achieve stable and robust operations, researchers have to develop many decision-making, autonomous navigation, and control algorithms [4,5,6].

In general, environmental understanding is the essential prerequisite for ensuring the stable and robust operations of autonomous robots [7], and many methods have been proposed up to now [8,9]. Optical camera-based methods have become very popular [10,11]. However, camera-based methods have several limitations such as the lack of geospatial and reflectivity intensity information, as well as image distortions and illumination variations. Consequently, traditional optical camera-based systems are difficult to be used for the understanding of unstructured environment [12].

In contrast, light detection and ranging (LiDAR) systems have been rapidly developed recently, which can obtain accurate geospatial and reflectivity intensity information [13,14]. Moreover, they are very robust to illumination variations and have much reduced image distortions. Therefore, LiDAR systems are more suitable for scene understanding and are gradually used in autonomous robots on unstructured environment [15,16,17]. Wang et al. proposed a fast plane segmentation algorithm to detect objects [18]. Díazvilariño, et al. used point clouds for detecting potential objects in the route planning according to the real state of the depictured obstacles [19]. However, the surface of unstructured terrain is uneven. Traditional planar-based segmentation methods cannot effectively deal with various shapes and different heights of obstacles on outdoor unstructured terrains [20].

Edges provide crucial information on terrain surfaces. Bazazian et al. proposed a fast and precise method to detect sharp edge features, which analyses the eigenvalues of the covariance matrix defined by k-nearest neighbors of each point [21]. Daniels et al. presented spline-based feature curves from point sampled geometry [22], and Oztireli et al. employed robust statistics to extract sharp features [23]. Lin et al. explored line segment extraction for large scale unorganized point clouds [24]. Wang and Feng employed the majority voting scheme to detect distinct geometric features such as sharp edges and outliers in a scanned point cloud [25], A region growing method that can segment the point cloud into clusters and identify the regions with sharp features was proposed based on the analysis of the normal of the points [26]. However, all these methods only perform well with sharp edges or edge features are particularly noticeable.

As extracting sharp edge features from a 3D point cloud requires accurate normal estimation, the performance of shared point-based techniques depends on the accuracy of the normal input, particularly for the relevant points located around the edge. Furthermore, normal estimation is computationally time-consuming for large scale point clouds. In this paper, a LiDAR system is deployed for an autonomous robot to understand the unstructured environment. The Sobel operator is applied to the edge detection of 3D laser point clouds, then optimizes the operator according to the characteristics of the 3D point cloud and realizes the edge detection of unstructured terrain.

The rest of the paper is organized as follows. Section 2 proposes a novel system framework for obstacles detection on unstructured terrain. Then, the obstacle feature recognition algorithm is presented in Section 3, which is based on LM-BP neural network. In Section 4, the proposed algorithm is firstly verified by using an unstructured terrain 3D mapping dataset from the Autonomous Space Robotics Laboratory (ASRL) of Canada. Section 5 conducts the experiments on a real robot platform to verify the proposed approach. Finally, a brief conclusion and future work are given in Section 6.

## 2. Materials and Methods

### 2.1. Introduction

**Figure 1:**
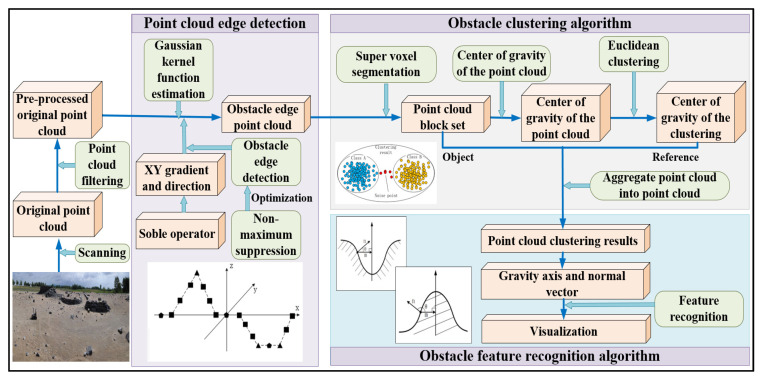
The proposed novel approach to obstacle detection in unstructured environment.

We propose a point cloud post-processing algorithm for obstacle detection based on an reconstructed unstructured terrain point cloud, which provides essential priori information for autonomous robots to operate in uneven and dynamic changing terrains. Figure 1 shows our proposed obstacle detection approach, which mainly includes three algorithms: (i) point cloud edge detection algorithm, (ii) obstacle-clustering algorithm with super-voxel segmentation, and (iii) obstacle feature recognition algorithm based on the LM-BP neural network. Note that algorithms (i) and (ii) will be explained in this section, and algorithm (iii) will be presented in the next section.

### 2.2. Point Cloud Edge-Detection Algorithm

The edge-detection operator consists of a first-order differential operator and a second-order differential operator. As the unstructured terrain point clouds contain a lot of noise, the first-order differential operator is used in this paper to reduce the influence of noise, which is also called the gradient operator. The gradient value of the image gray is maximum at the edge area, which is a vector and is expressed as:

(1) $$\nabla I\left(x,y\right)=\left(\frac{\partial I}{\partial x},\frac{\partial I}{\partial y}\right),\left|\nabla I\left(x,y\right)\right|=\sqrt{{\left(\frac{\partial I}{\partial x}\right)}^{2}+{\left(\frac{\partial I}{\partial y}\right)}^{2}}$$

(2) $$\theta =arctan\left(\frac{\partial I}{\partial y}\right)/\left(\frac{\partial I}{\partial x}\right)$$

where $\frac{\partial I}{\partial x}$ is the x-direction gradient. $\frac{\partial I}{\partial y}$. is the y-direction gradient. |∇I(x,y)| is the magnitude of the gradient, indicating the edge intensity information. θ is the gradient direction, providing trend information for an edge.

The first-order differential operators mainly include a Roberts operator [27], Sobel operator [28], Prewitt operator [29], and Canny operator [30]. The Roberts operator has high edge positioning accuracy but is sensitive to noise. The Prewitt operator suppresses noise by pixel averaging, but the edge positioning accuracy is underdeveloped. The Canny operator is complicated and easy to smooth out some of the edge information. In contrast, the Sobel operator introduces distance weights, thereby improving the ability to suppress noise, which therefore is used in this research.

**Figure 2:**
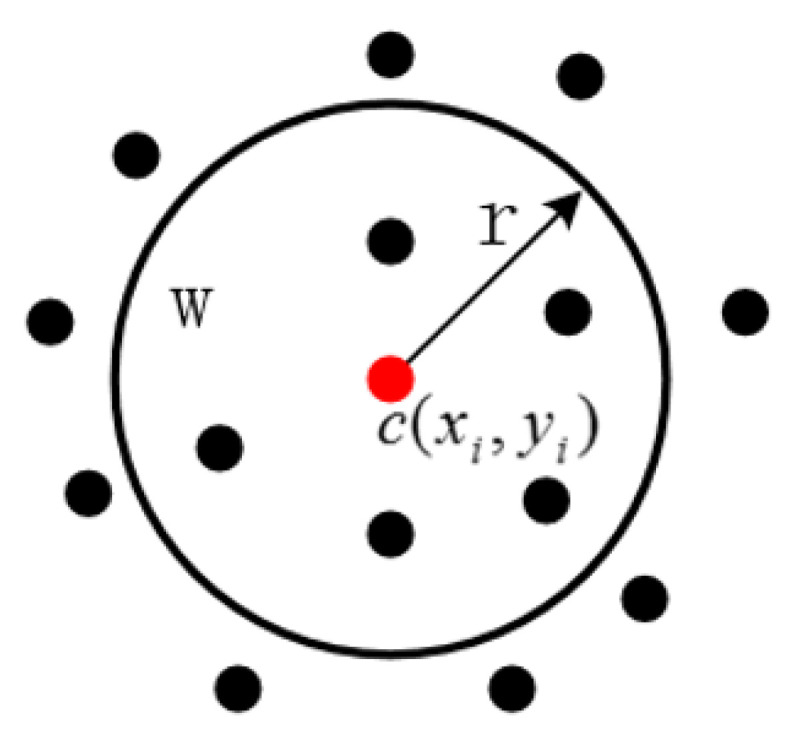
Gaussian kernel function estimation method.

Although the Sobel operator can effectively handle a 2D image whose pixels are evenly distributed, it cannot effectively handle the points of 3D point clouds that are mostly unevenly distributed in space, resulting in a large error. Therefore, the Gaussian kernel function estimation is introduced to solve this problem, which is shown in Figure 2. The point cloud is projected on to the plane. Point $c\left(x_{i},y_{i}\right)$ is the target point. The neighbor points of it in the *r* range are searched and then each neighbour point is used by the Gaussian kernel function. The *Z* value is weighted. Finally, the weighted mean is taken as the estimated *Z* value of the target point. The Gaussian kernel function used for weighting is:

(3) $$K\left(x_{w},y_{w}\right)=0.6171\exp\left(-\frac{1}{2}{\left(\frac{x_{w}-x_{i}}{2r}\right)}^{2}\right)\exp\left(-\frac{1}{2}{\left(\frac{y_{w}-y_{i}}{2r}\right)}^{2}\right)$$

where $\left(x_{w},y_{w}\right)$ is the neighboring point in the neighborhood of the target point r. $x_{i}$ and $y_{i}$ are the *x* value and the y value of the target point, respectively. *r* is the radius of the neighbour point search.

The weights of the neighboring points close to the target point are larger so that the accuracy of the target point z-value estimation is improved. For the target point $\left(x_{i},y_{i}\right)$, the estimated *Z* value is:

(4) $$Z\left(x_{i},y_{i}\right)=\frac{\sum_{\left(x_{w},y_{w}\right)\in w\left(c,r\right)}Z\left(x_{w},y_{w}\right)K\left(x_{w},y_{w}\right)}{\sum_{\left(x_{w},y_{w}\right)\in w\left(c,r\right)}K\left(x_{w},y_{w}\right)}$$

**Figure 3:**
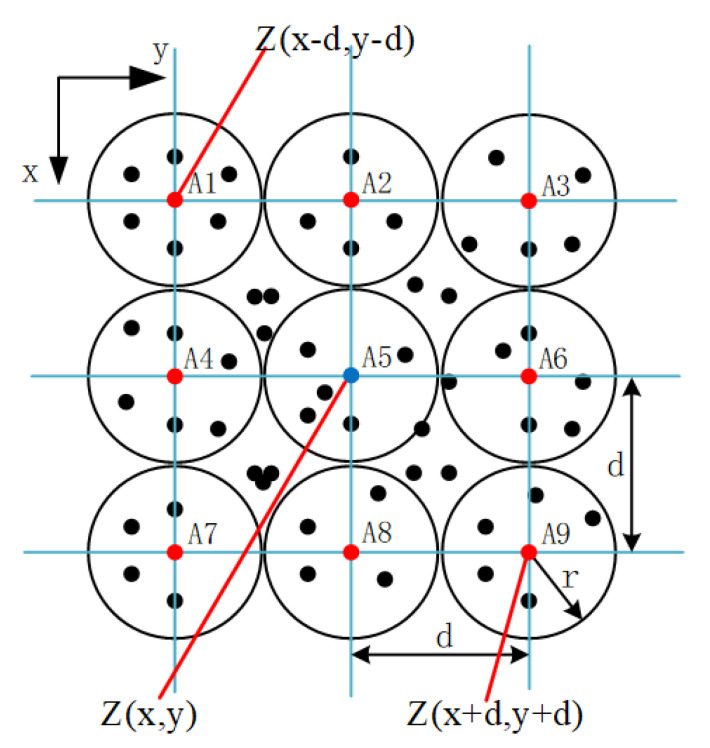
Sobel operator estimated by combining Gaussian kernel function.

As shown in Figure 3, the 3D point cloud is first projected onto the plane, in which A5 is taken as the target point of the estimated elevation gradient, *d* is the specified spacing and 8 neighboring points arranged in a planar grid array. The Gaussian kernel function estimation method is used to obtain *Z* of each neighboring point. Finally, the Sobel operator is used to calculate the elevation gradient of the target point.

**Figure 4:**
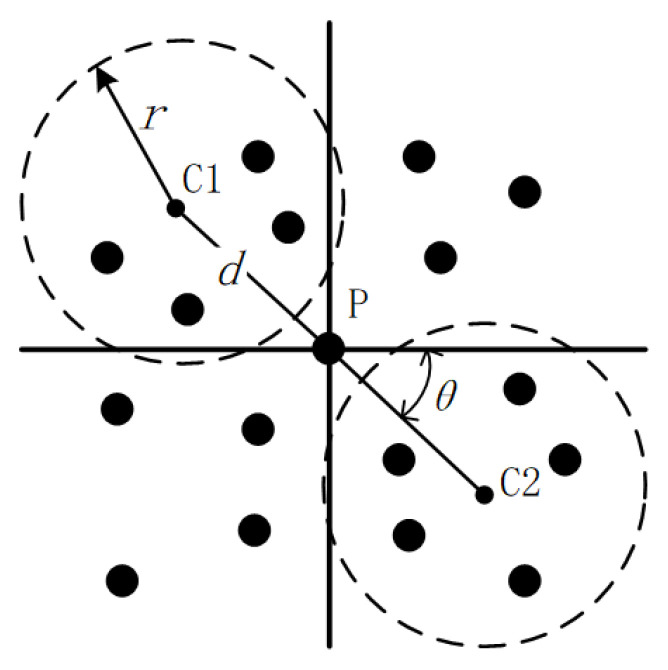
Non-maximum suppression applied to point clouds.

The edge detected by the Sobel operator contains a lot of redundant information. To further optimize the edge of the extracted obstacle and speed up the subsequent processing, a non-maximum suppression is utilized to eliminate elements that are not maxima in the local neighborhood. Figure 4 shows the implementation of the algorithm of the previously obtained obstacle edge point cloud, in which *p* is the target point and also the center of symmetry, *c*1 and *c*2 are the centers for search, r is the radius to search for the neighbors, *θ* is the angle along the horizontal direction, d is the distance between *c*1 and *c*2 in the gradient direction.

First, the point cloud is projected onto the plane, and then the gradient direction of the target point *p* is determined according to the previously calculated information. The elevation gradients of all the neighbors are taken as the elevation gradient values of *c*1 and *c*2 and compared with the gradient values of the target points. The target point is retained if both are smaller than the gradient value of the target point. Otherwise, it is rejected. After all the points in the point cloud have been processed as described above, an optimized obstacle edge point cloud is obtained.

### 2.3. Obstacle Clustering Algorithm with Super-Voxel Segmentation

Clustering is the process of dividing the similar data points into multiple independent point clouds such that the points in a point cloud are similar to each other but different from the points in other groups. The Euclidean clustering algorithm adopts the spatial distance between adjacent points as the criterion to judge whether the point clouds should be clustered into one group, as shown in Algorithm 1.
**Algorithm 1** Single Point Cloud Clustering**Input:** A point in point cloud (*P*)**Output:** Group of points (*Q*)Put *P* into *Q***while** (Points in *Q* has increased)Search for the KDTree nearest neighbor points *N* of *P***for each**$N_{1}\in N$**do****if** Distance from $N_{1}$ to *P* <= *Threshold*$N_{1}$ put into *Q***end if****end for**Select points other than point *P* in *Q***end while**

**Figure 5:**
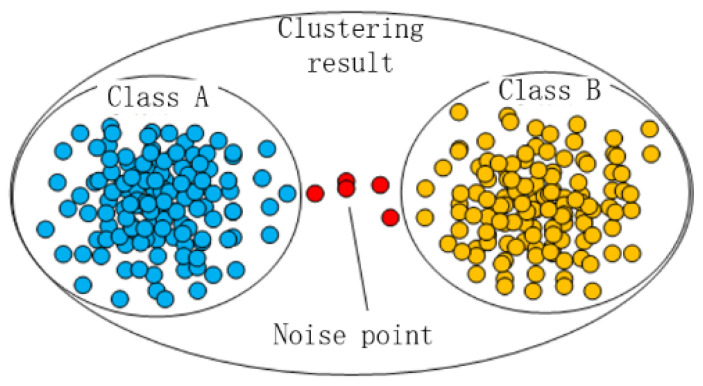
Effect of noise points on Euclidean clustering algorithm.

The Euclidean clustering algorithm can be implemented quickly, but has some limitations, such as initial guess of the number of groups/classes and a random choice of cluster centers which lack consistency. When there are some noise points, the Euclidean clustering algorithm is unable to achieve correct clustering. As shown in Figure 5, class *A* and class *B* should have been split. However, due to the influence of the noise points between the two clusters, they have been wrongly classified into a point cluster.

To solve this problem, a super-voxel segmentation method is introduced here to improve the anti-noise ability of the clustering process. It is a means of over-segmentation. According to the similarity of features the scene point cloud is divided into many small blocks for understanding the point cloud. The process is similar to the crystallization process. First, the crystal nucleus is uniformly arranged in the space after the point cloud data is voxelized, and then all the nuclei grow at the same time and similar particles (voxels) are continuously absorbed. Finally, the point cloud is segmented into a crystal, which is called the cloud block. Crystal growth is controlled by the following distance metric *D*:

(5) $$D=\sqrt{w_{c}D_{c}^{2}+\frac{w_{s}D_{s}^{2}}{3R_{seed}^{2}}+w_{n}D_{n}^{2}}$$

where $D_{c}$ is the difference in colour. $D_{s}$ is the difference in distance. $D_{n}$ is the difference in the normal direction. $w_{*}$ is the weight used to control the crystallization process. $R_{seed}$ is the nucleation distance.

The super-voxel segmentation method can make discrete noise points gather into small cloud blocks, which is convenient for filtering and improving clustering accuracy. At the same time, the gravity center of the point cloud block is used as a clustering object to improve the efficiency of the whole clustering process as shown in Algorithm 2.
**Algorithm 2** Obstacle point cloud clustering**Input:** Original point cloud (*O*)**Output:** Point cloud of obstacle clustering (C)Divide *O* into point cloud blocks (*B*) by super-voxel segmentation**for each**$B_{1}\in B$**do****if** point number of $B_{1}$ <= *Threshold*delete $B_{1}$**end if****end for**Calculate the gravity center of BCenter of gravity point cloud clustering

## 3. Obstacle Feature Recognition Based on Levenberg–Marquardt Back-Propagation (LM-BP) Neural Network

### 3.1. BP Neural Network Optimized by LM (Levenberg–Marquardt) Algorithm

The neural network has the self-learning function and can deal with incomplete, fuzzy, uncertain or irregular data. As the most widely utilized neural network, the BP neural network (BPNN) uses back propagation to repeatedly adjust the weights and bias of the network so that the output vector is extremely close to the expected vector [31]. However, it is easy to fall into the local minimum, as well as slow convergence and oscillations during training. Therefore, the LM (Levenberg–Marquardt) algorithm is used here to solve these problems, in which Gauss–Newton is used to generate an ideal search direction near the optimal value of the function approximation and the network weights are adaptively adjusted. Finally, the network convergence speed is greatly improved.

Let $w_{k}$ be the vector consisting of the weight and threshold of the kth iteration, then the weight of the (*k*+1)th is updated as:

(6) $$w_{k+1}=w_{k}+\Delta w$$

The weight update error index function E(w) is:

(7) $$E\left(w\right)=\frac{1}{2}\overset{N}{\underset{i}{\sum }}{\left(t_{i}-o_{i}\right)}^{2}=\frac{1}{2}\overset{N}{\underset{i}{\sum }}e_{i}^{2}$$

where *N* is the dimension of the output vector. $t_{i}$ is the target output of the ith output neuron in the output layer. $o_{i}$ is the actual output of the neuron.

For Newton’s method:

(8) $$\Delta w=-H_{k}^{-1}g_{k}$$

where $H_{k}$ is the Hessian matrix of the error index function E(w) and $g_{k}$ is the gradient.

(9) $$g=J^{T}\left(w\right)e\left(w\right)$$

(10) $$H=J^{T}\left(w\right)J\left(w\right)e\left(w\right)+S\left(w\right)$$

(11) $$S\left(w\right)=\overset{N}{\underset{k=1}{\sum }}e_{i}\left(w\right)\nabla^{2}e_{i}\left(w\right)$$

where $e\left(w\right)={\left[e_{1}\left(w\right),e_{2}\left(w\right),\cdots ,e_{N}\left(w\right)\right]}^{T}$ and J is the Jacobian matrix.

(12) $$J=[\begin{array}{cccc} \frac{\partial e_{1}\left(w\right)}{\partial w_{1}} & \frac{\partial e_{1}\left(w\right)}{\partial w_{2}} & \cdots & \frac{\partial e_{1}\left(w\right)}{\partial w_{n}}\\ \frac{\partial e_{2}\left(w\right)}{\partial w_{1}} & \frac{\partial e_{2}\left(w\right)}{\partial w_{2}} & \cdots & \frac{\partial e_{2}\left(w\right)}{\partial w_{n}}\\ & & \;\vdots & \\ \frac{\partial e_{N}\left(w\right)}{\partial w_{1}} & \frac{\partial e_{N}\left(w\right)}{\partial w_{2}} & \cdots & \frac{\partial e_{N}\left(w\right)}{\partial w_{n}} \end{array}]$$

When the minimum value of the energy function is approached, the element value of the matrix S(w) becomes extremely small. Therefore, the Hessel matrix is:

(13) $$H\approx J^{T}\left(w\right)J\left(w\right)$$

(14) $$\Delta w=-{\left[J^{T}\left(w\right)J\left(w\right)\right]}^{-1}J^{T}\left(w\right)e\left(w\right)$$

The LM algorithm is used to improve the Gauss–Newton method, which overcomes the inconsistency of the network caused by the instability of Gauss–Newton inversion in the Hessel matrix. The LM algorithm is obtained by modifying Formula (13).

(15) $$H\approx J^{T}J+uI$$

where *u* is an extremely small number and *I* is an n×n identity matrix.

The LM network weights are updated to:

(16) $$w\left(k+1\right)=w\left(k\right)-{\left[J_{k}^{T}\left(w\right)J\left(w\right)+uI\right]}^{-1}J\left(w\right)e\left(w\right)$$

**Figure 6:**
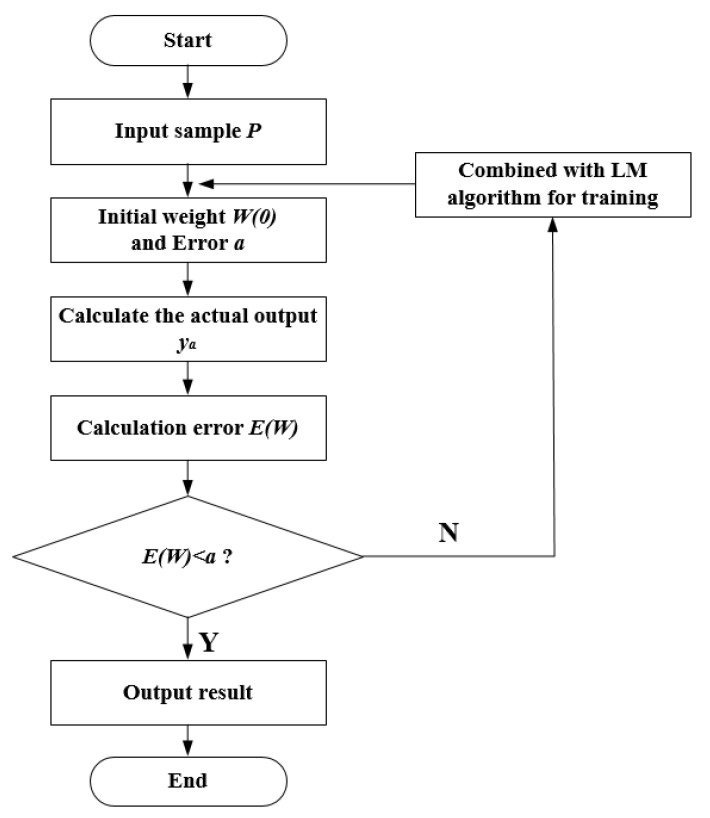
The flowchart of the Levenberg–Marquardt (LM) algorithm to improve the back-propagation (BP) neural network.

Figure 6 shows the flowchart of the LM algorithm to improve the BP neural network.

**Figure 7:**
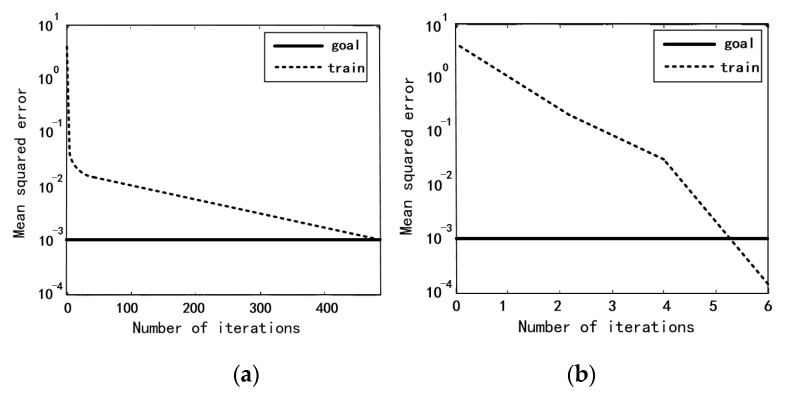
Comparison of network training curves. (**a**) Original BP neural network training curve; (**b**) LM algorithm-improved BP training curve.

Figure 7 shows the original BP neural network training curve and the LM algorithm-improved BP neural network training curve. As can be seen, the convergence speed of LM-BP neural network training has been significantly improved.

### 3.2. Feature Selection and Evaluation Indicators

**Figure 8:**
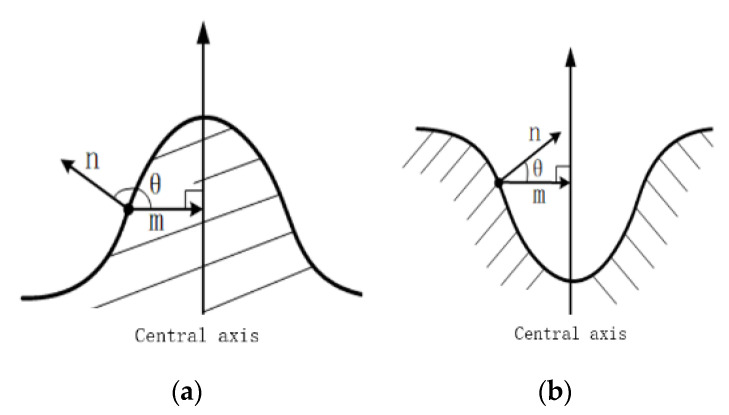
Profile analysis of positive and negative obstacles. (**a**) positive obstacle; (**b**) negative obstacle.

Figure 8 shows the profile analysis of obstacles, in which $\vec{n}$ is the normal vector of a surface point of the obstacle, $\vec{m}$ is the horizontal vector pointing from the surface point to the central axis, and *θ* is the angle between the two vectors. For a positive obstacle, *θ* is an acute angle. For a negative obstacle, *θ* is an obtuse angle. 3D obstacles have innumerable sections that produce numerous central axes. Therefore, the central axis is replaced by the gravity axis center of an obstacle in practical applications. At the same time, the *θ* corresponding to all surface points of the obstacle (all points in the single obstacle point cloud) is calculated as one of the main features of the positive and negative of the target obstacle.

**Figure 9:**
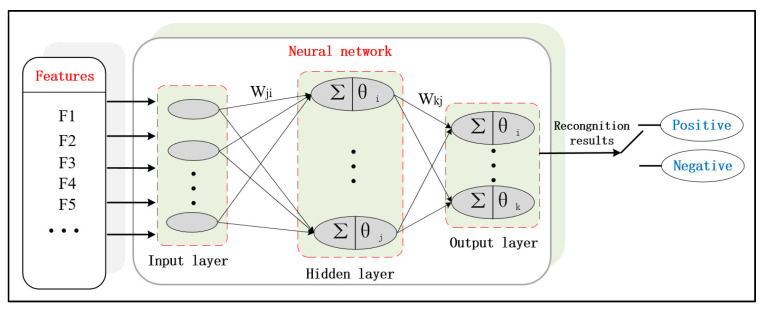
Structure of the established neural network.

Finally, both geometric and dimensional features are used to separate the different characteristics of obstacles: (i) F1: the average height; (ii) F2: maximum height; (iii) F3: minimum height; (iv) F4: the number of acute angle θ; (v) F5: the number of obtuse angle θ. Based on this feature, the structure of the established neural network for recognizing positive and negative obstacles is shown in Figure 9.

To quantitatively assess the accuracy and correctness of our classification method, three metrics are employed, namely *recall*, *precision* and *F1-measure*. The *recall* represents the percentage of true positives in the ground truth, the *precision* represents the percentage of true positives in the extracted result, and the *F1-measure* is a combination of the two metrics. They are calculated as follows:

(17) $$recall=\frac{TP}{TP+FN}$$

(18) $$precision=\frac{TP}{TP+FP}$$

(19) $$F1\;measure=\frac{2\cdot recall\cdot precision}{recall+precision}$$

where *TP*, *FN* and *FP* denote the number of true positives, false negatives and false positives respectively.

## 4. Experimental Verification on Dataset

**Figure 10:**
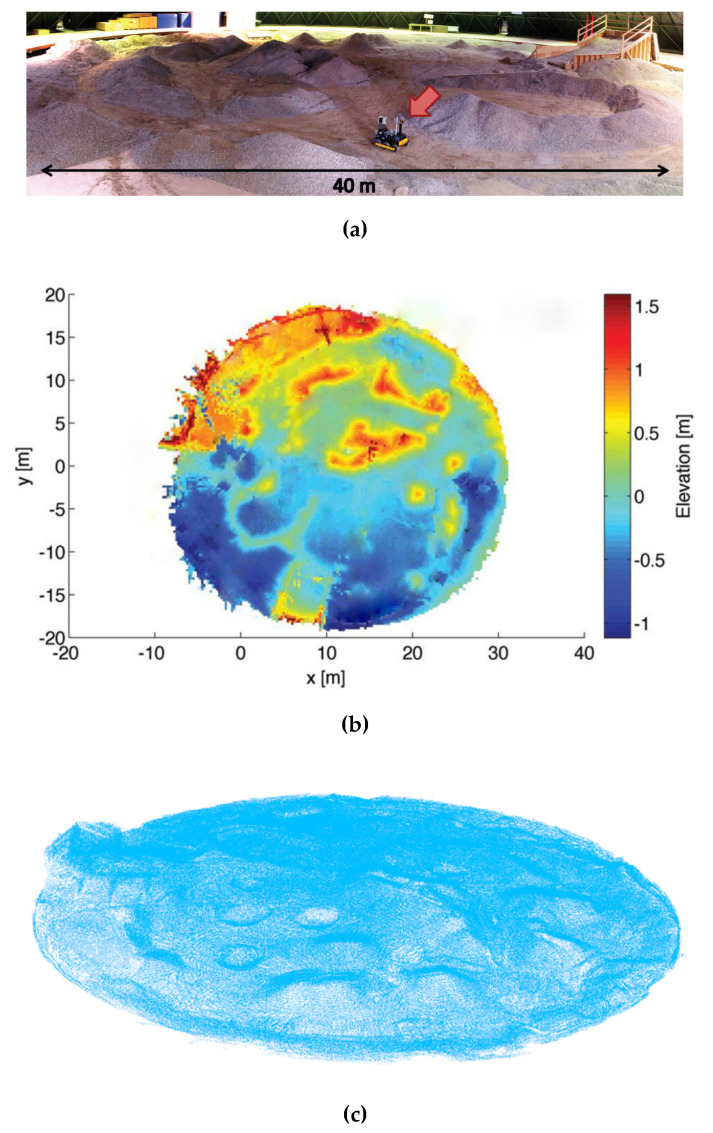
The unstructured terrain 3D mapping dataset. (**a**) Original picture of the unstructured terrain; (**b**) depth image of the unstructured terrain; (**c**) point cloud of the unstructured terrain (914608 points).

The proposed algorithm is firstly verified by experiments on the 3D mapping dataset of an unstructured terrain from the Autonomous Space Robotics Laboratory (ASRL) of Canada [32], as shown in Figure 10. Many researchers conducted their research on robotic navigation and obstacle avoidance based on this unstructured terrain data set [33,34,35]. We also use it in the experiments to verify the proposed algorithm.

### 4.1. Obstacle Edge-Extraction Experiment of Terrain Point Cloud

**Figure 11:**
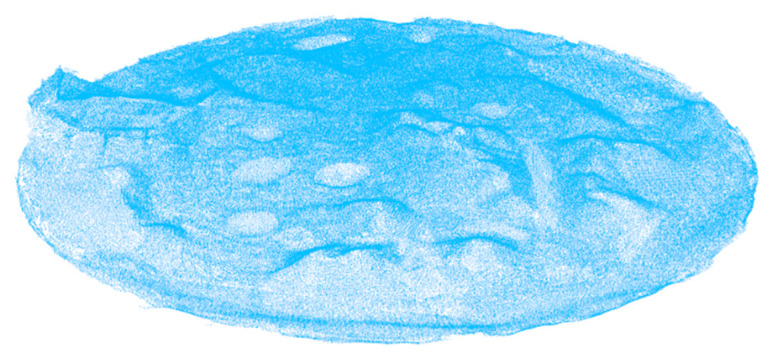
Unstructured terrain point cloud after filtering (413591 points).

**Figure 12:**
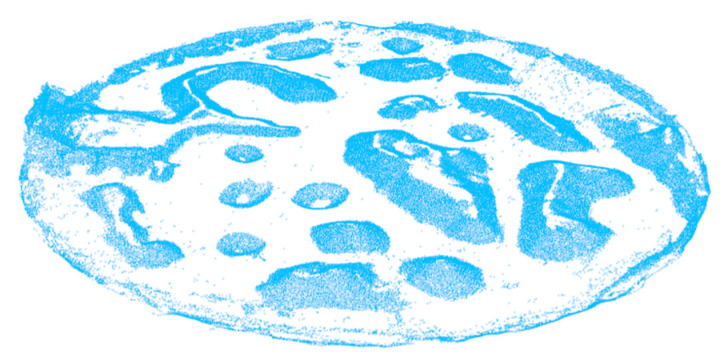
Results of obstacle extraction based on edge detection (82242 points).

**Figure 13:**
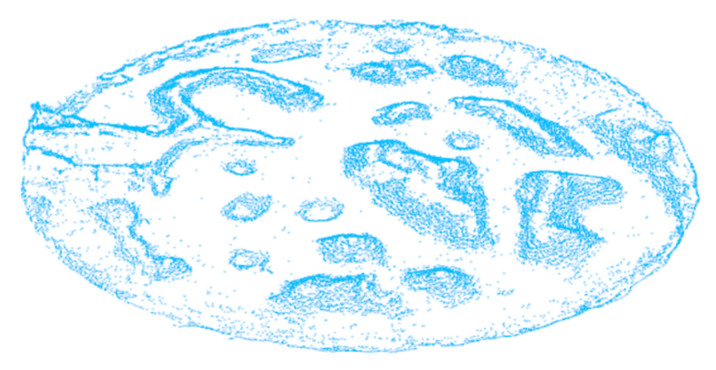
Optimization of obstacle extraction based on non-maximum suppression (26011 points).

Figure 11 shows the point cloud (413591 points) generated after the filtering method. The data is streamlined, and the noise is reduced to provide a good foundation for the following obstacle extraction experiments. Figure 12 shows the results of the obstacle extraction experiment based on edge detection. As can be seen, the ground points of the non-obstacle in the topographic point cloud are filtered out and the same is the point of the flat area at the top of obstacle. Therefore, the shape and contour of the extracted obstacle are completely clear. The useful information is completely preserved and enhanced while most of the redundant information and noisy data are eliminated. Figure 13 shows the optimization result of obstacle extraction based on non-maximum suppression (26011 points).

### 4.2. Obstacle Clustering Experiment of Terrain Point Cloud

**Figure 14:**
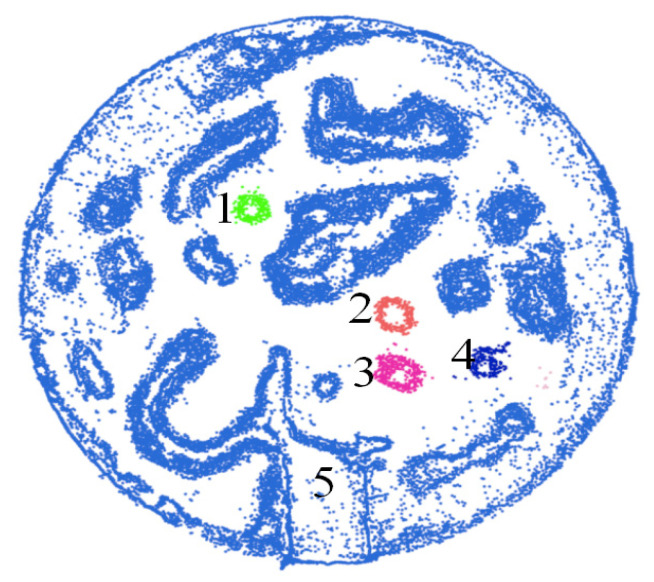
Results of direct use of the Euclidean clustering method.

**Figure 15:**
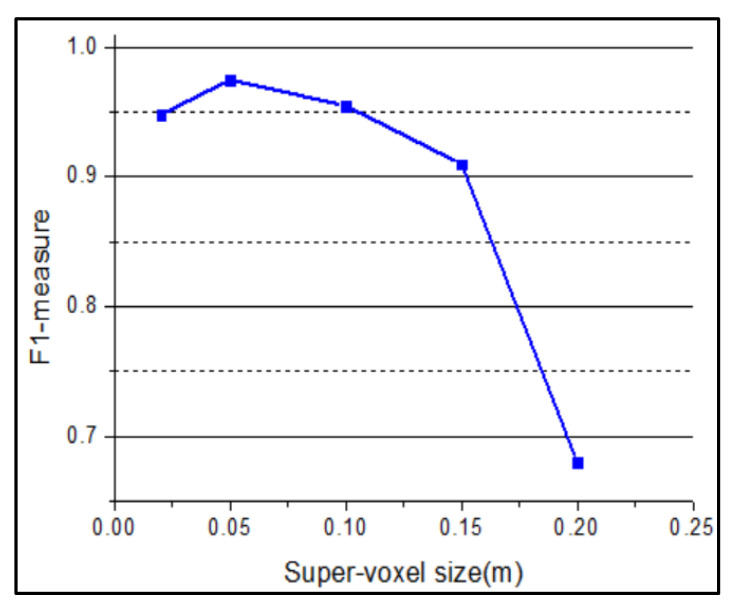
F1-measure achieved by proposed method with different sizes of super-voxel.

To verify the effectiveness of the obstacle-clustering method combined with super-voxel segmentation, the Euclidean clustering algorithm is used to cluster the obstacle point cloud clusters. The clustering results are shown in Figure 14. The obstacle point cloud is divided into five categories, and the phenomenon of under-segmentation appears. Many different obstacles are divided into the same class (Category 5) due to the influence of a large number of scattered noisy points. The different sizes of the super-voxel are firstly tested as our method is based on the super-voxel. Figure 15 shows the F1-measure performance of the proposed algorithm under different super-voxel sizes. As a result, we choose the super-voxel size as 0.05 m.

**Figure 16:**
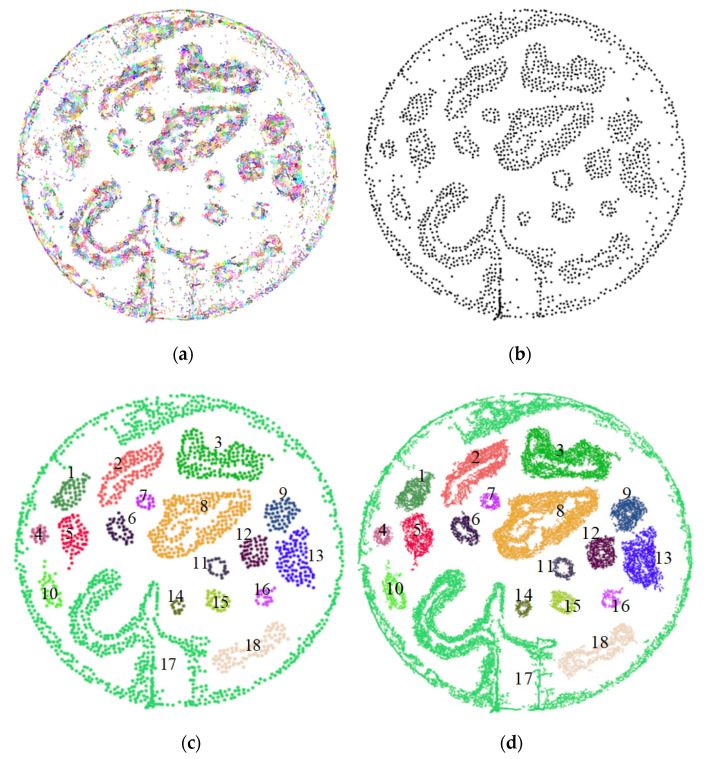
The process of clustering obstacles combined with super-voxel segmentation. (**a**) Super-voxel segmentation results; (**b**) center of gravity point cloud; (**c**) center of gravity point cloud clustering results; (**d**) obstacle point cloud clustering results.

Figure 16 shows the process and results of clustering experiments using the obstacle-clustering method combined with super-voxel segmentation. More specifically, Figure 16a shows the results of the super-voxel segmentation of the original obstacle point cloud, in which different point clouds are distinguished from each other in different colors. It can be seen that the obstacle point cloud is divided into small units and relatively sparse and independent noise forms a small cloud. By culling these small point clouds (points <6) and calculating the gravity center of the points in the remaining point-cloud blocks, the point cloud of the gravity center is shown in Figure 16b. It can be seen that most noises in the point cloud have been filtered out and the cloud of each obstacle is further highlighted, which provides a positive initial condition for the Euclidean clustering method.

Figure 16c shows the clustering results of the center of the gravity point cloud, in which different point clouds are given different colors. In the end, the point cloud was split into 18 point clouds, and the noise was further suppressed by setting the minimum number of cluster points. However, the 17th group is not completely divided as the convex plate is connected to the top of the terrain. The other point clouds of the obstacle achieved an excellent clustering segmentation effect. Figure 16d shows the obstacle point-cloud clustering results, in which the point cloud block is aggregated. In comparison with the clustering results of Figure 14, Figure 16d show the effectiveness of the obstacle-clustering method combined with super-voxel segmentation. It not only achieves complete separation of individual obstacles, but also suppresses a large amount of noise, making the extracted single obstacle data more accurate.

### 4.3. Experiment of Recognising Obstacles on Terrain Point Clouds

**Figure 17:**
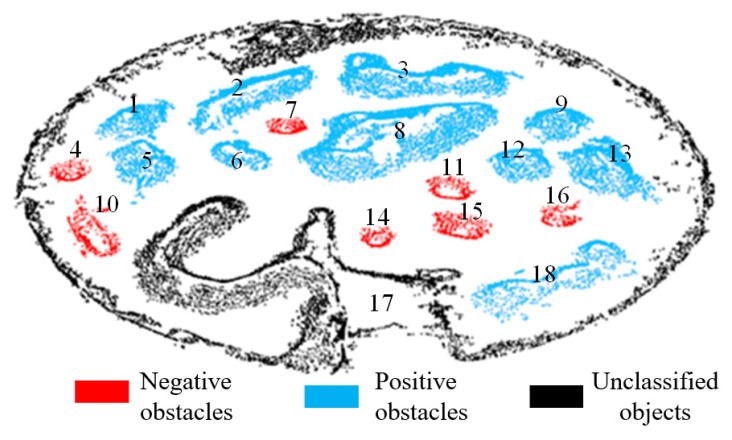
Obstacle recognition result of the 3D mapping dataset.

Figure 17 shows the obstacle recognition result from the 3D mapping dataset of an unstructured terrain [32]. As the 17th point cloud is not completely divided and is not processed, it is marked as black. For the remaining obstacle point cloud clusters, the positive obstacle is marked in blue and the negative obstacle is marked in red. As can be seen in Figure 17, the point clouds 4, 7, 10, 11, 14, 15 and 16 are judged as negative obstacles, and the rest are positive obstacles, which matches the actual situation.

## 5. Experimental Verification on Real Unstructured Terrain

### 5.1. System Configuration

**Figure 18:**
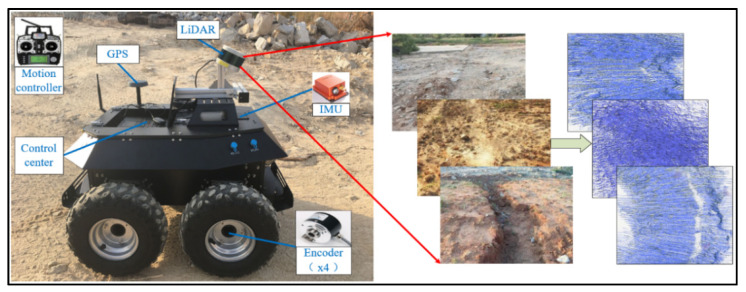
Mobile light detection and ranging (LiDAR) point-cloud data acquisition system.

Figure 18 presents our proposed framework, in which a LiDAR (Velodyne VLP-16, which produced by Velodyne Lidar of San Jose, California, U.S.) is mounted on an all-terrain robot for acquiring the point cloud data. Table 1 shows main parameters of the sensors fixed on the mobile platform. As there are redundancy and uncertainty among the used sensors with different sampling frequencies, we use the multi-sensor data fusion technology to improve the reliability and robustness of the system.

**Figure 19:**
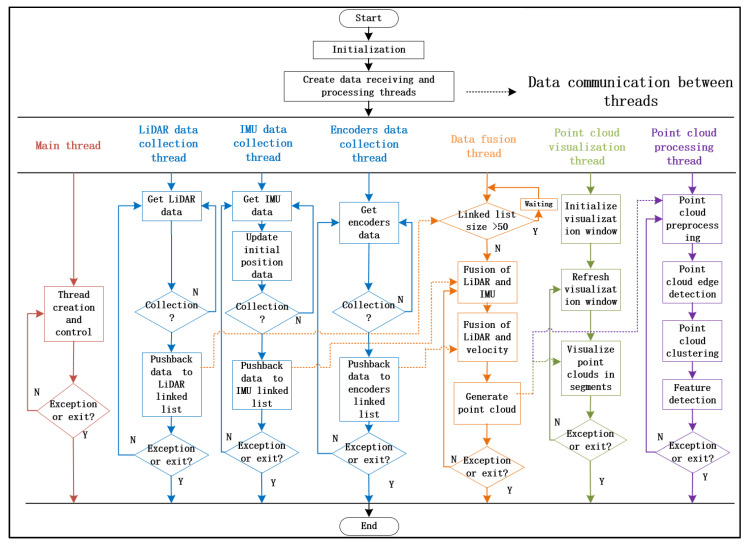
The flowchart of the multi-sensor data fusion algorithm based on multi-thread technology.

Figure 19 shows the flowchart of our multi-sensor data fusion algorithm, which takes advantage of the parallelism of multi-threading to achieve efficient collection and processing of multi-sensor data. Moreover, its flexibility and extensibility ensure that the system can be efficiently supplemented.

### 5.2. Real Experimental Results

**Figure 20:**
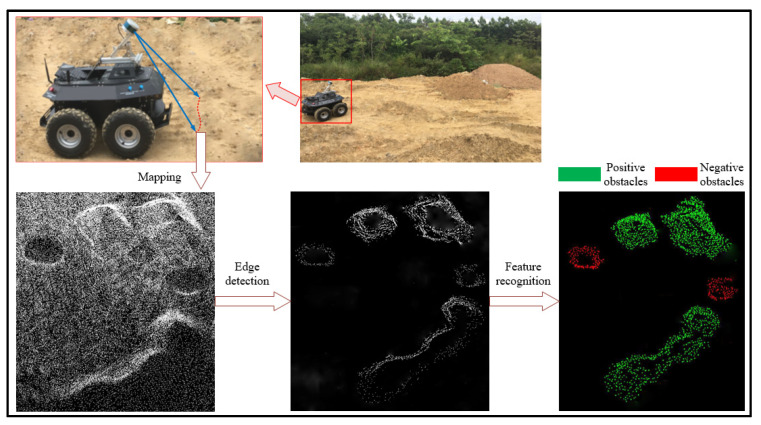
Results for obstacles detection on real unstructured terrain point cloud.

**Figure 21:**
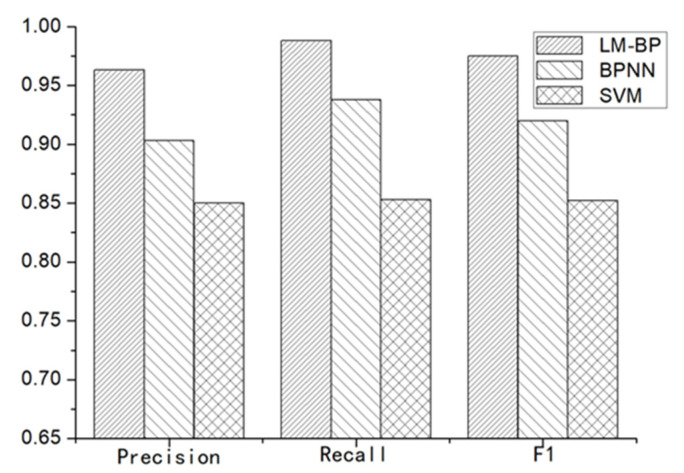
Accuracy comparison for obstacle recognition using Levenberg–Marquardt back-propagation (LM-BP), back-propagation neural network (BPNN) and support vector machine (SVM).

To further verify the performance of the proposed algorithm, we used an all-terrain robot and reconstructed the real unstructured terrain point cloud as the original point cloud based on a two-step registration algorithm [36]. Figure 20 shows the experimental results, which clearly show that the proposed approach can effectively detect positive and negative obstacles within the unstructured terrain. We collected 65 frames of the original terrain point clouds with 325 positive and negative obstacles to compare the proposed algorithm with the traditional BPNN algorithm and SVM (Support vector machine) algorithm. Figure 21 shows the comparison experiment results which clearly show that our approach outperformed the traditional BPNN algorithm and SVM algorithm. The *precision*, *recall* and *F1-measure* of proposed algorithm are 0.963, 0.988 and 0.975 respectively, which can be effectively used to detect and recognize obstacles on unstructured terrain.

## 6. Conclusions

This paper presents a novel laser-based approach for obstacle detection of autonomous robots. The Sobel algorithm and the Gaussian kernel function estimation are deployed to effectively handle the points of 3D point clouds for the extraction of obstacle edges in the unstructured terrains. Then, a non-maximum suppression method is introduced to optimize the result of obstacle extraction. Furthermore, super-voxel segmentation is combined with the Euclidean clustering algorithm to achieve robustness and high-precision clustering segmentation of obstacle point clouds. Finally, a LM-BP neural network is created to recognize the positive and negative obstacles. Both the existing dataset and a real unstructured terrain point cloud reconstructed by an all-terrain robot are used to verify the proposed point cloud post-processing approach. The results of extraction, clustering and recognition have demonstrated the effectiveness of the proposed approach. Out future research will be focused on further practical applications such as path planning and obstacle avoidance of autonomous robots in an unstructured environment.

## Figures and Tables

**Table 1:** The main parameters of the sensors.

Sensors	Model	Physical Data	Main Parameters
LiDAR	Velodyne VLP-16	3D Point of terrain	Measurement range: 100 m. Accuracy: ±3 cm. Angular Resolution (Horizontal): 0.1°–0.4°. Angular Resolution (Vertical): 2.0°.
IMU (Inertial measurement unit)	Xsens MTi-700	Euler angle	Latency: <2 m. Bias repeatability: 0.1°/s. Sampling frequency: 10KHz.
Encoder	E6B2-CWZ6C	Velocity	Accuracy: 1000 P/R; Maximum speed: 6000 r/min. Maximum response frequency: 100 KH.
